# An Unusual Cause of Spontaneous Pneumomediastinum: The Mounier-Kuhn Syndrome

**DOI:** 10.1155/2019/5359309

**Published:** 2019-07-08

**Authors:** Salim Naciri, Rachida Zahraoui, Mouna Soualhi, Jamal-Eddine Bourkadi

**Affiliations:** Moulay Youssef Hospital, Pulmonary Unit, University Mohammed V of Rabat, Morocco

## Abstract

Mounier-Kuhn syndrome is a rare clinical and radiologic condition. It is characterized by tracheal and bronchial dilation. Diagnosis is made by computed tomography and bronchoscopy. An 81-year-old man presenting with an acute chest pain was referred to the pulmonology department. His chest computed tomographic scan showed a tracheobronchomegaly with an increase in the diameter of both the trachea and right and left main bronchi, associated with pneumomediastinum and fibrosis. Fiberoptic bronchoscopy revealed enlarged trachea and both main bronchi. These findings are consistent with a diagnosis of Mounier-Kuhn syndrome. Besides considering this long-neglected “orphan disease” when diagnosing spontaneous pneumomediastinum, clinicians should also be aware of an underlying Mounier-Kuhn syndrome in patients with recurrent respiratory infections, in order to avoid complications associated with the disease.

## 1. Introduction

Mounier-Kuhn syndrome (MKS) or congenital tracheobronchomegaly is a rare clinical and radiologic condition. It is characterized by tracheal and bronchial dilation [1]. Histology shows loss of the main airway smooth muscle and cartilage and an associated tracheal diverticulosis, but its etiology remains uncertain. Dilation of the trachea and proximal bronchi is associated with a decreased mucociliary clearance, airway inflammation, inefficient cough and bronchiectasis, and/or emphysema [2]. Rare cases of spontaneous pneumothorax have been reported, but only one case was related to a spontaneous pneumomediastinum.

## 2. Case Presentation

An 81-year-old man presenting with an acute chest pain was referred to the pulmonology department. He had no smoking history but recurrent respiratory infections.

The physical examination revealed that the patient was in good general health, with tachypnea at rest. The examination of patient's chest revealed the presence of bilateral rales, more on basal regions.

Labs were unremarkable except for arterial blood gas that noted a decline in respiratory function (PaO2=63mmHg) (Table 1).

**Figure 1:**
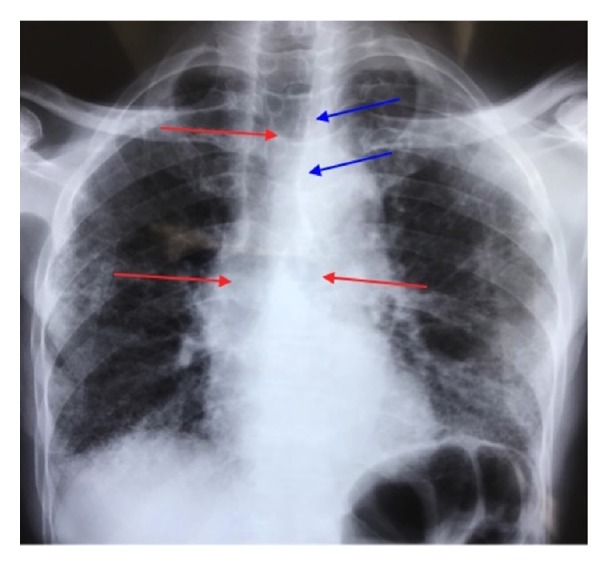
Chest X-ray showing tracheal and proximal bronchi dilation (red arrows) and pneumomediastinum (blue arrows).

**Figure 2:**
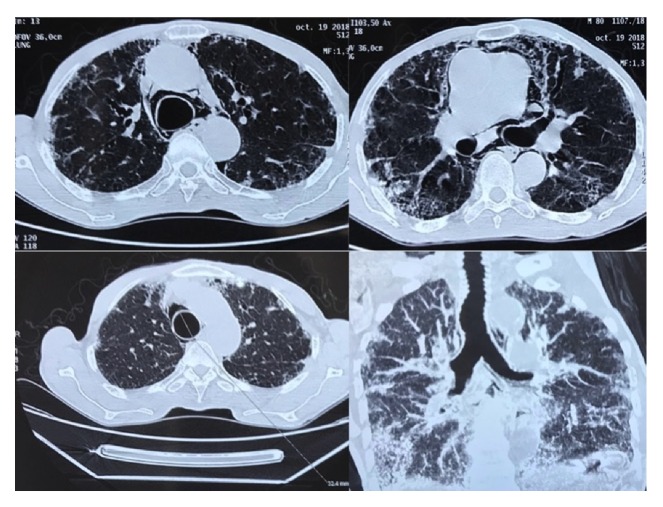
CT scan showing tracheal and both main bronchi dilation with associated pneumomediastinum.

**Figure 3:**
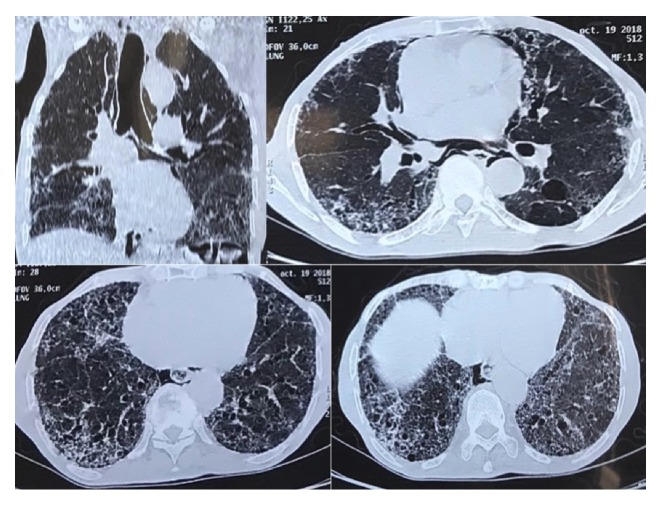
CT scan showing bronchiectasis, bullous emphysema, and fibrosis.

Enlargement of the trachea and a pneumomediastinum were detected in the chest radiograph (Figure 1). The chest computed tomographic (CT) scan showed tracheobronchomegaly with an increase in the transverse diameter of the trachea and right and left main bronchi measured at 32, 26, and 25 mm, respectively (Figure 2). Bronchiectasis was noted in the bilateral lungs, bullous emphysema was noted in the bilateral upper lobes, and fibrosis was detected in the bilateral lower lobes (Figure 3).

Fiberoptic bronchoscopy revealed tracheal dilation and enlargement of both main bronchi.

The bronchoscopy findings coupled with the findings on CT chest confirmed a diagnosis of MKS.

**Figure 4:**
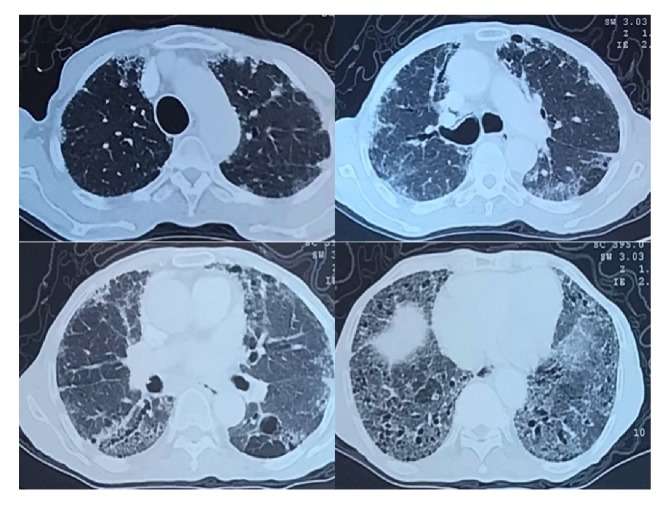
Control CT scan showing regression of the pneumomediastinum.

The patient made good progress with high flow oxygenotherapy administered for 8 days, and the pneumomediastinum remitted in the control CT scan (Figure 4). The patient was discharged and followed up in an outpatient setting. The arterial blood gas control (Table 1) confirmed the decline in respiratory function, and pulmonary function testing revealed moderate airflow obstruction.

## 3. Discussion

The syndrome was first described by Mounier Kuhn in 1932. It is characterized by a dilation of the trachea and main bronchi due to laxity to the walls of the airway with decreased mucociliary clearance [2, 3]. This leads to formation of diverticula and bronchiectasis and recurrent infections. The prevalence is relatively low, affecting between 1% and 4.5% of the population and generally presents in the third or fourth decade of life [4–7]. There is a strong male predominance (about 8:1) [6, 7] and most of the patients seem to be smokers. Non-smokers have also been reported [2] like our presentation of an 81-year-old man non-smoker with a long history of respiratory symptoms.

Radiologic diagnosis can be established with plain chest radiograph alone [2] but CT has become the golden standard for confirming the diagnosis [8] by giving abnormal airway measurement (increase in the tracheal transverse diameter, right and left main bronchi upper than 3 cm, 2,4 cm, and 2,3cm, respectively, in adults) and may additionally demonstrate associated pathology. Krustins' analysis of 128 published cases between 1987 and 2013 revealed the presence of tracheal diverticulosis in 33% of patients with this syndrome [9]. However, our patient's CT scan revealed enlargement of trachea and both main bronchi and also bronchiectasis, emphysema, and pulmonary fibrosis.

Although some patients have no smoking history, as in our case, the association with COPD is frequently reported in 48,5% over 33 patients who had lung function test results in Krustins' study [9]. The relationship between MKS and COPD remains unclear.

Most frequent pulmonary complications quoted in literature are bronchiectasis (49,2%), bullous emphysema, recurrent pneumonia, and aspergillosis [9–12]. Fibrosis and decline in respiratory function are also described in some reports like a series of eleven cases [13] which reported complications as bronchiectasis in 54% and one case of parenchymal fibrosis, as in our presentation. Another case of fibrosis complicating the MKS was also described in an eight-case series [7].

We are aware of only two cases presenting initially with spontaneous pneumothorax [5, 14], and to the best of our knowledge, only one research in the literature has reported MKS being a possible cause of spontaneous pneumomediastinum [15]. The case reported by Simkins was about a 95-year-old man with spontaneous pneumomediastinum and pneumonia.

The most likely pathophysiological mechanism involved in the genesis of pneumomediastinum in our case would be the existence of a decreasing pressure gradient between the alveoli and the lung interstitium that can result in alveolar rupture. Another possible explanation for pneumomediastinum is the abnormal increase of pressure in the mediastinum, like in a context of vigorous coughing, for example, that could cause the rupture of tracheal diverticula, frequently encountered in MKS [13]. In the case described by Simkins, the mechanism was related to oesophageal perforation [15].

The described case report offers a rare insight into the natural course of the progression of an undiagnosed MKS. Factors affecting disease progression in patients have not been studied; therefore the prognosis of patients with MKS is largely unknown [9].

This case supports early diagnosis in patients with MKS so that a decline in respiratory function resulting from possible complications of MKS may be prevented.

## 4. Conclusion

Mounier-Kuhn syndrome or tracheobronchomegaly is a very rare condition whose congenital or acquired origin is still controversial. The fact that clinical signs are not very specific should be considered as a differential diagnosis in patients with pneumomediastinum, even if the association is very rare as our case is the second reported in literature. The radiological diagnosis is easy, based on a careful analysis of the central airways and pulmonary parenchyma by CT examination. However, more studies have to be carried out to understand the etiology and natural course of this orphan disease.

## Figures and Tables

**Table 1:** Patient blood results.

Blood test	Blood count	Serum electrolytes	Others
Results	Hemoglobin=14,2g/dL	Na+=138mmol/L	Liver enzymes, urea, creatinine: without any particularities
	White Cells=5490 elements/mm3	K+=4,20mmol/L	
	Platelets =247000/mm3	Glucose=1,0 g/L	
		C Reactive Protein=7	

Blood test	Arterial blood gas	Arterial blood gas control	

Results	pH=7,39	pH=7,4	
	PaO2=63mmHg	PaO2=65mmHg	
	PaCO2=44mmHg	PaCO2=42mmHg	
	SaO2=93%	SaO2=94%	

## Abbreviations

CT: Computed tomography
MKS: Mounier-Kuhn syndrome
COPD: Chronic obstructive pulmonary disease.
